# The Nursing Supplement

**Published:** 1889-12-21

**Authors:** 


*The Hospital*, December 21, 1889.

### The Nursing Mirror.

*Extra Supplemen*

All Contributions for this Supplement should be addressed to the Editor, The Hospital, 139-140, Salisbury Court, Fleet Street, London, E.C., and should have the word "Nursing" plainly written in left-hand top corner of the envelope.

### En Passant.

A MERRY CHRISTMAS.—To all our readers far and near—A Merry Christmas! To our correspondents in Australia and America, in India and on the continent, we send our very best wishes at this festive season. There are some of our readers from whom we have heard so often they have become our friends, and we ever hope to maintain these friendly relations which are so pleasant a break amongst mere business details. Hospitali lfe is particularly hard at Christmas time, and our sympathy is with all those nurses throughout the kingdom who are striving to cheer the patients under their charge, and who add to their nursing labours the duty of decorating wards and singing carols. And those private nurses who are now parted from their friends, and at this time of general merry-making have to keep watch beside the bed of some patient who is seriously ill, or maybe dying, have also our sympathy, and our admiration for their courage and devotion to duty. But even to them we can wish a happy Christmas and a new year full of pleasure and of those rewards which follow surely on all earnest labour; for "the working of the good and brave endures for ever."

CHRISTMAS ENTERTAINMENTS.—Such merry-making as is consistent both with the season and with duties to the sick is being carried on in most of the hospitals. At the London Hospital, a nurses' conversazione was given by Mr. and Mrs. Carr-Gomm last night (Friday), and a Christmas-tree will be held in the children's ward on the afternoon of December 31. At St. Bartholomew's, theatricals are in active rehearsal; and, on January 2 and 3, scenes from "The Critic" and "The Merchant of Venice" will be played in the great hall. There will also be minor entertainments in the different wards. At the Hospital for Sick Children, Great Ormond-street, there will be theatricals, etc., on December 27 and 28, and the small patients will have Christmas-trees each in their own wards, as large crowds are apt to introduce epidemics into children's hospitals. At Charing Cross there were theatricals on December 18th, 19th, and 20th, and Golding ward has already arranged for a little concert of its own on January 1st. At St. Thomas's, preparations are also in progress, but at Middlesex—poor Middlesex!—there are neither plans nor preparations. Several of the infirmaries are preparing Christmas amusements, and so are numerous provincial hospitals. We hope to give accounts of a few of their entertainments next week.

NURSES FOR WAKEFIELD.—The Misses Reed and Drew, certified trained nurses, late of the Institute for Trained Nurses, Leeds, have opened a nursing institute at 64, Northgate, Wakefield. They will supply trained nurses, and will shortly be in a position to take in resident patients who can have their own doctors.

NEW HOME AT MIDDLESBROUGH.—It is proposed to establish a nurses' home at Middlesbrough. For some time past one of the leading local medical gentlemen has, in conjunction with members of his family, engaged the services of a lady who is a trained experienced nurse for work among his own patients, and the experiment has been so successful that it is now proposed to make what has hitherto been a private and limited undertaking a general and unlimited one.

LECTURES FOR NURSES.—Last week Dr. R. H. Humphreys gave an excellent lecture at 15, Buckingham-street, on "Phlegmasia Dolens." There were only between 20 and 30 nurses present, and, remembering that the club has now 200 members, this number seems strangely small. Dr. Humphreys began by giving the premonitory symptoms which he thought it well a nurse should know, that she might warn her patient what was coming on. He then described the appearance of a limb thus affected, dwelling on the indiarubber-like feeling when pressed. The cause of phlegmasia dolens, the fact that it is due to the plugging of the lymphatics rather than to obstruction of a single vein, and the treatment generally resorted to, were points dwelt on. The lecture was both instructive and interesting, and illustrated by diagrams.

SHORT ITEMS.—Miss Thorn, who was elected matron at Ayr when Miss M'Ewen resigned, has been specially thanked by the committee and by the house surgeon for the way in which she performs her duties.—Two nurses are already at work at King's Cross Hospital, Dundee, under Miss Ramsay. It is expected that six nurses will be needed in all. The new uniform for this hospital is a dark blue cotton with narrow white stripe.—Miss Alice Stone, lecturing at Montreal, expresses her surprise at the dearth of nurses in Canada.—Miss Atkinson and Miss Darling were admitted to the Red Cross Sisters' Nursing Home at Dublin on December 4.—Lady Lewisham has started nursing lectures at Kirkburton.—Mrs. Curr's trustees will give £1,000 to the Dundee Sick Poor Nursing Society.—Sister Taylor sang so well at the anniversary concert at Jaffray Hospital that she was encored. Amongst the audience was Miss Bettney, matron.—Mrs. Musgrave's lectures on "Home Sick Nursing," at Edinburgh, are very interesting and well attended.—Five of the Sisters of Bethany are going from St. Agnes', Kennington, to the mission in Kurdistan.—There being eight cases in Wimbledon Fever Hospital, a second nurse has been engaged.

ET CETERA, ET CETERA.—The following is a specimen of the style of journalism indulged in by a contemporary. In a leader last week on the meeting of the General Medical Council it quoted as follows: "It was moved by Sir John Simon, seconded by Dr. MacAlister, and agreed to, 'That in the opinion of the council it would be much to the advantage of the public, and particularly would be of much convenience to the practitioners of medicine and surgery, that facilities, usable under proper guarantees in all parts of the United Kingdom, should be given, by Act of Parliament or otherwise, for the authoritative certification of competent trained nurses, who when certified should be subject to common rules of discipline,' etc., etc." The "etc., etc.," consisted of the following lines, which are such a severe blow to the writers in the journal in question, we do not wonder at their wish to suppress them, but only at their audacity in thus trying to delude their readers: "but that it does not appear to be within the province of the council to propose legislation or other action for the object referred to, nor has the council had any occasion to consider in detail the means by which the object might best be attained; and that, under these circumstances, the council cannot give any opinion on the particular questions asked of it in paragraph 5 of the memorandum of the British Nurses' Association." Special attention is called to the article on the Registration of Nurses, and to the memorandum of the B. N. A. to the General Medical Council, published in another column of the present issue.

### Notes on Opiates.

*(From the Note Book of a Nurse who attended Dr. Symes Thompson's lectures.)*

It is much to be feared that opium-taking is greatly on the increase. One mode of taking it is hypodermically—that is, injecting one or more drops of concentrated solution of morphia under the skin by means of a small syringe armed with a hollow needle, and specially designed for the purpose. The advantage of this plan is that it does not produce the disagreeable effects of nausea and headache as does taking it by mouth, and used subcutaneously less than half the quantity is required. When administered hypodermically the effect is very potent, but anyone who gets into the habit of thus taking it is obliged to continually increase the dose in order to produce the same effect, and if suddenly the dose is stopped the patient does not necessarily suffer. Sometimes when pain is very great it is partly justifiable, but it is too often used at very slight provocation, and when the habit is once established the craving for it is almost irresistible. The following is De Quincey's description of the effect on himself of opium-eating :

"I have thus described and illustrated my intellectual torpor in terms that apply, more or less, to every part of the four years during which I was under the Circean spells of opium. But for misery and suffering, I might, indeed, be said to have existed in a dormant state. I seldom could prevail on myself to write a letter, an answer of a few words to any that I received was the utmost that I could accomplish, and often that not until the letter had lain weeks, or even months, on my writing-table. Without the aid of M. all records of bills paid or to be paid must have perished, and my whole domestic economy . . . must have gone into irretrivable confusion . . . It is one, however, which the opium-eater will find in the end as oppressive and tormenting as any other, from the sense of incapacity and feebleness, from the direct embarrassments incident to the neglect or procrastination of each day's appropriate duties, and from the remorse which must often exasperate the stings of those evils to a reflective and conscientious mind. The opium. eater loses none of his moral sensibilities or aspirations ; he wishes and longs as earnestly as ever to realise what he believes possible and feels to be exacted by duty, but his intellectual apprehension of what is possible infinitely outruns his power, not of execution only, but even of power to attempt. He lies under the weight of incubus and of nightmare ; he lies in sight of all that he fain would perform, just as a man forcibly confined to his bed by the mortal languor of a relaxing disease, who is compelled to witness injury or outrage offered to some object of his tenderest love ; he curses the spells which chain him down from motion ; he would lay down his life if he might but get up and walk, but he is powerless as an infant, and cannot even attempt to rise."

Not only is self-control lessened, but even moral feeling and the sense of truth, letting those who have been reliable become entirely unreliable ; they break up their houses, thinking people speak evil against them, and imagine all sorts of wrongs are being done to them, and they lose all love for and confidence in even their dearest friends, and in these respects resemble some forms of insanity. Another peculiarity of these people is, that they become very angry if their supply of opium is kept from them.

Opium, chloral, and morphia have all very much the same effect; but chloral is the least objectionable, because it does not cause nausea, and brings sleep more easily and pleasantly than opium does, and if it be taken only once or twice it does no special harm, but if the dose be increased it is dangerous and seductive. Nurses who have access to sedative draughts, like medical men who dispense their own drugs, are very apt to take sedatives to procure sleep or relieve pain, thinking little of the nature or source of the evil. Thus a habit is formed most injurious in its consequences, and often very difficult to overcome. Because it has no immediate ill effects people think they can take it with impunity, but if it is persevered in it causes an eruption on the face, redness of throat, impairs the eyesight, makes deglutition difficult, and, if continued, in spite of all these symptoms idiocy will ensue.

Bromide is not so dangerous as opium, and does not so markedly pervert the moral sense and destroy truthfulness. It is often useful in cases of epilepsy ; epileptic patients are very apt to become morose, and to think themselves misunderstood, and, in addition, the disease tends to destroy memory, with very sad results in some cases.

Morphia, injected hypodermically, is very rapidly absorbed, and should only be used under medical advice, as it is dangerous under some circumstances, especially if there is any kidney disease. The habit—which is now only too common—of injecting morphia without medical advice is very much to be regretted. There is no doubt that where there is much worry a stray dose of opium is very useful, as it brings sleep ; or a pleasant state of reverie and a capacity for sleep.

*(To be continued.)*

### The Nurse's Bookshelf.

### MEDICINE IN INDIA.*

Now that so many nurses are going out to India, we feel it is well to bring to their notice a manual which may there be of the greatest use to them. There are certain points connected with sanitation and hygiene in India with which an English nurse is not familiar ; and there are certain fevers and epidemics there met with which are exceedingly rare here. On all points such as these Sir William Moore gives clear and concise directions, and as the book is arranged alphabetically throughout, and also contains an excellent index, it is easy to turn at once to the subject on which information is required. The language used is simple, as few technicalities as possible being introduced, and there are plenty of diagrams and illustrations. There is no necessity to praise a book which is in the fifth edition, but we wish specially to point out its usefulness to a nurse who is going to India for the first time.

### MINOR SURGERY AND BANDAGING.†

This useful little book of Mr. Heath's has long been a favourite with many trained nurses, and if old copies are worn out they had better be replaced by this new edition, which contains the latest methods of bandaging, and some new hints on anæsthetics. The book is one specially written for house surgeons, but we are sure nurses will be none the worse for noting its sensible opening chapter on the relations between the house surgeon and the matron and nurses. Mr. Heath says nurses have two chief faults : 1st, a desire to do all the dressings themselves, to save trouble ; and 2nd, a tendency to make favourites of certain patients to the neglect of others. The book is illustrated, has an index, diet tables, and a few common formulæ : it is indeed an admirable handbook for an house surgeon or dresser.

* "A Manual of Family Medicine and Hygiene for India." By Sir William Moore, K.C.I.E. Churchill. Fifth edition.

† "Minor Surgery and Bandaging." By Christopher Heath, F.R.C.S. Ninth edition. Churchill.

### Notes from Australia.

(*By our own Correspondent*).

MELBOURNE, Oct. 8.

WE are getting quite learned over here about your English hospitals, for Mr. J. Williams has just returned from his tour of inspection. His heart seems to have been won by the Marylebone Infirmary, and he advises the authorities of Melbourne Hospital to undertake reconstruction on a similar plan. He says: "The Marylebone Infirmary is under the control of the Local Government Board. The area of the site is 4a. 8p., about the same as the Melbourne Hospital reserve. This building is considered one of the most perfect of the class. Fireplaces of novel construction are placed in the centre of the wards. These connect with air channels passing under the floor. An efficient system of lifts is provided, and a home for nurses. By the kindness of the lady superintendent, I have been furnished with a detailed description of the building, which was constructed at a cost of £175 per bed, while the Edinburgh Infirmary cost £477, St. Thomas's £777, and the Hôtel Dieu £1,215 per bed. A most noticeable feature in all the large hospitals is the attention which is given to the department of nursing. Lady superintendents, or matrons who have had special training in every branch of their duty, occupy the post of head of the nursing staff. Ranking among the first in importance is the Nightingale Fund School, at St. Thomas's Hospital. Accommodation is provided for a large number of probationers, and a branch of the same school is carried on at the Marylebone Infirmary. Probationers are divided into two classes—(*a*) the ordinary probationer, who eventually take the duty of nurse, (*b*) the special probationers, who train for future heads of hospitals or departments of hospitals." It is probable that Mr. Williams's suggestion will be carried out. Mrs. M'Kie, the matron of the Melbourne Hospital, has lately resigned on account of ill-health, and it is hoped that a thoroughly-trained and efficient matron will be appointed, who will be able to raise the level of nursing in the hospital. Money is coming in to the Increased Hospital Accommodation Fund, and soon, I hope, I shall be able to send you more cheerful news about our medical charities.

A new convalescent home for women has been opened in the Clayton-road.

The Central Board of Health held a meeting lately. The president stated that during the week 39 cases of diphtheria had been reported to the board from various parts of the colony, 16 of which had resulted fatally. The total number of deaths from diphtheria from January to July of the present year was 185, as against 82 for last year, and 40 for 1887. The president also said that a paper had been written by Dr. Shields on the treatment of diphtheria in the absence of a medical man. It would be printed, and submitted to the board at its next meeting. He had been informed that a mixture of carbolic acid and glycerine had been found a very beneficial remedy in cases of diphtheria.

Our Medical Act is likely to be amended soon. A deputation waited on the Attorney-General, and Dr. Youl, as spokesman, said the object of the deputation was to ask for an alteration in the Medical Registration Act of this colony by the repeal of the clause which provided that no person should be registered in this colony unless he were a naturalised or natural born subject. The striking out of two or three words would make all the alteration required. The Medical Council of England refused to register persons from this colony on account of the state of the law here.

Some articles on the Melbourne slums which have been appearing in the *Argus* have been much talked over here. They show that vice is as rampant in this colony as in the old country, but they also show how marvellous is the charity of ladies who keep open all-night shelters and otherwise work at women's mission to women.

Another subject of importance is a powerful article on immigration, called "The Broader Duty of Australia." The writer compares Australia, nineteen-twentieths wilderness crying for cultivators, and the seething mass of humanity in London slums. He maintains that the selfish party is now in power in Victoria, and fear to introduce immigrants in case their own wages be cut down by competition. He urges that it is our duty as citizens of the world to endeavour to induce England's capital and England's labour to come here together. Truly there is a beautiful country here waiting for folks to claim it.

### Everybody's Opinion.

[*Correspondence on all subjects is invited, but we cannot in any way be responsible for the opinions expressed by our correspondents. No communications can be entertained if the name and address of the correspondent is not given, or unless one side of the paper only be written on.*]

### CERTIFICATES AND REGISTRATION.

MISS HEANLEY writes: Much has been said and written lately on this subject. It is necessary first to test the value of certificates as granted by various hospitals, those with a recognised training school, lectures, and examinations, and those where the practical training is as good, but there are no lectures, and no "machinery" for examination, where the probationers must read for themselves, assisted by the matron, and by the clinical information given by the medical men. It might appear on the face of it that these latter probationers must suffer in comparison and take a lower stand than those trained in larger hospitals. I say, not necessarily, and that a general examination for all is the only fair test for all, both for certificates and for registration. How make these certificates valuable, and sufficient, with a certain length of practical service (say, as has been suggested, three years), as a pass to legal registration? Would it not be easy to follow on the lines of the St. John's Ambulance Association regarding the examinations of their local classes? Let each county hospital act as a centre for examination to the hospitals in that county; each large training school to the smaller hospitals in its district. Let the examinations be conducted by disinterested examiners, appointed on a "Hospitals' Examination Staff," like the examiners of the St. John's Society. Let each examinee pay a fee towards defraying the expenses of the examinator. Let a certain standard of efficiency be fixed by the heads (doctors and matrons) of the recognised successful training schools. Could this not be done amicably, all desiring, as I am sure they all do, the ultimate raising of the standard of trained nursing to one universal level? Could we not thus surmount the difficulty of long journeys, straining the often slender resources of our nurses' pockets, which must occur if they are obliged to go to London, Edinburgh, Dublin, Manchester, Liverpool, or Birmingham for their examinations? As regards our midwives, they must have passed the obstetrical examination, and so stand on their own lines for registration; but even they should have at least three years' practice before becoming eligible, and they would be all the better, too, for a year's training in general hospital work, for knowledge is always power, and never to be despised. As for monthly nurses, these two points, a year's general hospital training and three years' practice, should be a *sine qua non* for granting them registration. What do women with six weeks, or even three months' training know of the nursing which may be required of them in the unforeseen complications that sometimes accompany child-birth; do such nurses, as a rule, understand how to pass a catheter or give enemas? They do not. As a London head midwife told me, "No, we do not consider it needful to teach those things, nor have we the time to give them general training."

### Notices.

### HELP WANTED FOR A CRIPPLE.

Can anyone tell me how to get £18 a year to keep a well-educated, superior woman (not a lady) out of the workhouse? She is terribly crippled by rheumatism, etc., and unable to earn her living.—Nurse V. Those willing to help should communicate with the Editor of The Hospital, The Lodge, Porchester-square, W.

### EXHIBITION OF NURSING UNIFORMS.

Through an error the name of Nurse Brickler was given as the dresser of the National and Metropolitan doll which won a prize; the name should have been Miss Bertha Berry. The uniform of the Devonport Royal Albert Hospital was described as black instead of navy blue; but really in these dark days in London such mistakes are bound to occur. Amongst the dolls which were highly commended but not mentioned last week were London Hospital (Miss Porter), Newport and County Hospital, National Aid Society, and the South London Nursing Association. We once more repeat that the dolls, after being shown in the provinces, will find a final resting place in a nurses' museum in London.

### Appointments.

[It is requested that successful candidates will send a copy of their applications and testimonials, with date of election, to The Editor, The Lodge, Porchester-square, W.]

Monsall Hospital, Manchester.—(*Lady Superintendent*).—Miss Florence Calvert has been appointed lady superintendent of nurses to Monsall Fever Hospital, Manchester. Miss Calvert trained at Guy's, and afterwards was appointed "sister" at St. Saviour's Infirmary, Dulwich, where she remained seven months, during that time filling both the night superintendent's and assistant matron's places during holidays. Miss Culvert left Dulwich owing to her appointment as night superintendent at Monsall Hospital, which post she filled from October, 1888, till the end of November, 1889, when she obtained her present post. Miss Calvert has the reputation of being well fitted to fill this responsible post, and her many friends in the nursing will be pleased of her rapid promotion.

Monsall Hospital, Manchester.—(*Night Superintendent*).—Miss Esther Jones has been appointed night superintendent of Monsall Fever Hospital, Manchester. Miss Jones trained at the Royal Southern Hospital, Liverpool, and was afterwards sister at Cardiff Infirmary for some time. She has been a sister at Monsall Hospital for over two years.

Cyprus Society.—Miss Christian, who trained at St. Bartholomew's, is going to Cyprus in January as nurse to the above society.

Aldershot Lock Hospital.—Mrs. Richardson, who was for several years head nurse at Chatham Lock Hospital, has been appointed matron of the Lock Hospital at Aldershot.

### Notes and Queries.

To Correspondents.—1. Questions may be written on post-cards. 2. Advertisements in disguise are inadmissible. 3. In answering a query please quote the number. 4. If a private answer is desired, a stamped addressed envelope must be forwarded.

Notice.—We must really ask our correspondents to be more particular in enclosing name and address. Constantly we receive queries or letters with no name attached.

### Queries.

(34) *Books on Massage.*—Who are the publishers of Weir Mitchell's book on massage? Has Sir Lyon Playfair written a treatise on the subject?

(36) *Nursing the Lepers.*—I should be glad of any information concerning the Government appointments for nurses in the leper hospitals about to be established in India. *L. A. D.*

### Answers.

*Monthly Nursing.*—Four weeks, counting by the day of the week, is the usual time given by a monthly nurse.

*Chatelaines.*—Private nurses can wear these or not as they choose. If they don't wear them, they must carry scissors, etc., in their pockets.

*Masseuse.*—The best way of obtaining the books you want cheaply, would be to advertise in our exchange column. Write to the publisher about it.

### Death in our Ranks.

On December 3rd was buried Mrs. Mary Harcus, for 25 years nurse in the Miners' Hospital at Marske. Amongst those present at the funeral were Sir J. W. Pease and the Ven. Archdeacon Yeoman. The coffin was carried by 12 miners who had been under the loving care of Mrs. Harcus while she was a nurse. Her death is greatly regretted in the village, where she was always ready to minister to the sick and suffering.

### Vacancies.

The following vacancies are announced:

### Matrons.

Bath Nurses' Institute, £50.
Kent County Asylum (assistant), £85.
St. Alban's Hospital.
Swindon G. W. R. Hospital, £35.
Tyrone Infirmary, £50.

### Night Superintendent.

Queen's Hospital, Birmingham, £30.

### Nurses.

Barony Parochial Hospital, Glasgow, £24.
Bedfordshire Institute, £26.
Birmingham and Midland Skin Hospital.
Blackheath Institute.
Bournemouth Institute, £25.
Bridgwater Infirmary, £25.
Brixton Nursing Institute.
Central London Throat Hospital, £20.
Chester Deaconess Institute, £24.
City of London Hospital, Victoria Park, £24.
Colchester Hospital, £35.
Eastbourne Institution.
Frome Union, £25.
Hampstead Home Hospital, £20.
Hanwell Ophthalmic School, £26.
Jersey Nurses' Institute, £25.
Kent Nursing Institution.
Leicester Institution, £25.
London and Brighton Association.
Loughborough Infirmary.
Macclesfield Infirmary, £24.
Middlesbrough Fever Hospital, £30
Newport Nursing Institute.
North-Western Fever Hospital, £30.
Nursing Institution, 96, 97, 98, Wimpole-street, London, W., Mr. Wilson has several vacancies for thoroughly-trained nurses.
Portsmouth Infectious Diseases Hospital (two).
Sheffield Nurses' Home.
South-Eastern College, Ramsgate, £30.
Southport Nurses' Home.
South Wales Nursing Institute (several).
St. George's Infirmary, Fulham-road.
St. Helena Home, N.W.
St. Olave's Union, S.E. (several).
St. Saviour's Infirmary, £20.
Truro Infirmary, £20.
Workhouse Infirmary Nursing Association.

### District Nurses.

Coventry Nursing Association, £25.

### Probationers.

Boston Hospital.
Canterbury Nurses' Institute.
Kensington District Association.
Liverpool Hospital for Women.

### Amusements and Relaxation.

**Third Quarterly Word Competition Commenced October 5th, 1889, ends December 28th, 1889.**

Three Prizes of 15s., 10s., 5s., will be given for the largest number of words derived from the words set for dissection.

Proper names, abbreviations, foreign words, words of less than four letters, and repetitions are barred; plurals, and past and present participles of verbs, are allowed. Nuttall's *Standard* dictionary only to be used.

N.B.—Word dissections must be sent in WEEKLY, arranged alphabetically, with correct total affixed.

The word for dissection for this, the *Twelfth* week of the quarter, being "MISTLETOE."

### Results of Tenth Week.

| Names. | Dec. 12th. | Totals. | Names. | Dec. 12th. | Totals. |
|---|---|---|---|---|---|
| Winifred | — | 672 | Sunshine | 45 | 1195 |
| Adelaide | — | 646 | Electrician | 48 | 1339 |
| Nettie Vance | — | 757 | H. C. | — | 562 |
| F. W. P. H. | 48 | 1388 | Reldas | 43 | 1396 |
| Amos | — | 596 | Rover | — | 776 |
| Reyot | — | 369 | Qu'appelle | 37 | 1131 |
| Ellice | 45 | 1414 | Beryl | — | 188 |
| A. S. K. | — | 275 | Lightowlers | 48 | 1440 |
| Neptune | 38 | 1314 | Hercules | — | 12 |
| Ecila | 45 | 1472 | Jenny Wren | 44 | 1005 |
| Tinie | — | 17 | Night Watch | — | 346 |
| Amicus | — | 946 | Oakenrod | 48 | 1170 |
| Juvenis | — | 539 | Embryo | — | 385 |
| Jumps | 48 | 1468 | Gypsye | — | 967 |
| Sister | — | 438 | Leo | 23 | 699 |
| M. W. | 48 | 1412 | Pearl | — | 299 |
| Esperance | 48 | 1411 | Africando | — | 171 |

### Notice to Correspondents.

N.B.—All letters referring to this page which do not arrive at 139 and 140, Salisbury-court, London, E.C., by the *first post on Thursdays*, and are not addressed PRIZE EDITOR, will in future be disqualified and disregarded.

